# Observation of a Fano Resonance at 92 meV (13.5 µm) in Al_0.2_Ga_0.8_N/GaN-Based Quantum Cascade Emitters

**DOI:** 10.3390/mi16070787

**Published:** 2025-06-30

**Authors:** Daniel Hofstetter, Andreas D. Wieck, Hans Beck, David P. Bour

**Affiliations:** 1 Independent Researcher, Chemin du Château 5, 2068 Hauterive, Switzerland; 2 Lehrstuhl für Angewandte Festkörperphysik, Ruhr-Universität Bochum, Universitätsstrasse 150, 44780 Bochum, Germany; andreas.wieck@rub.de; 3 Independent Researcher, Rue des Peupliers 6, 2014 Bôle, Switzerland; hans.beck39@bluewin.ch; 4 Google LLC, 1250 Reliance Way, Fremont, CA 94539, USA; davidpbour@gmail.com

**Keywords:** Fano resonance, Al_0.2_Ga_0.8_N/GaN structure, quantum-cascade emitter, LO-phonon energy, inter-subband transition

## Abstract

We report on asymmetrically shaped Fano resonances in Al_0.2_Ga_0.8_N/GaN-based quantum cascade structures. In order to observe this type of resonance in electro-luminescence, a spectrally narrow feature must interact with a broad, quasi-continuous emission. While the narrow waveform is provided by the GaN-based LO-phonon at 92 meV (13.5 µm, 741 cm^−1^), the broad peak consists of overlapping inter-subband transitions between several higher-order excited states ranging from 80 to 300 meV and the ground state. Through the interference of these spectrally dissimilar peaks, a typical, asymmetric Fano line shape is generated.

## 1. Introduction

The earliest experimental evidence of an optical phenomenon involving interference effects between a sharp, discrete energy level and a broad, quasi-continuous spectrum dates back to the year 1902 [1]. This paper was published by Robert W. Wood in the journal ‘Philosophical Magazine’, and it did not actually contain any formulas—just several photographs and schematic drawings. Nevertheless, it showed the typical asymmetric line shape of what would later be known as Fano resonance. Shortly before a more detailed theoretical description of this particular resonance by the Italo–American physicist Ugo Fano [2], John J. Hopfield [3] and Hans J. Henning [4] reported similar experimental observations as Robert W. Wood almost three decades earlier [1]. In early 1935, the German experimental physicist Hans Beutler published in the journal ‘Zeitschrift für Physik’ [5] a paper on asymmetrically broadened spectral lines of heavy noble gas atoms such as [Ar], [Kr], and [Xe] interacting with a continuous [He] discharge. A more detailed theoretical explanation of these results followed shortly thereafter. This eminent paper on the topic had been written in Italian by Fano later in that (same) year, 1935, and it appeared in the scientific journal ‘Il Nuovo Cimento’ [2]. It then took another couple of years until the terms ‘Fano interference’ and ‘Fano resonance’ started to be used for the typical asymmetric line shape that had been observed.

As much as 26 years after Fano’s early theoretical work, it was again Ugo Fano who published in 1961 a more comprehensive study on generally the same topic. His review article can be found in reference [6]. Although the fascinating physics of this very comprehensive treatment continued—along with the earlier publications from the 30s—to gather a certain academic interest, it took quite a while until this work was recognized by a broader scientific audience [7,8,9]. Many years after these pioneering papers, the resonant interaction of optical phonons with two-dimensional electron and hole space-charge layers on silicon has been identified as a Fano resonance as well [10]. The triggering factor for yet increased attention was, without any doubt, the demonstration of modern photonic crystal (PC) structures [11]. Since the base material of these PCs was, in general, a high-quality semiconductor crystal, the discrete energy levels of its LO-phonons could happen to resonantly interact with one of the quasi-continuum PC energy levels. There are finally some reports of Fano interferences in nanostructures and metamaterials, like in a paper by Luk’yanchuk [12], in a work about resonant-grating filters by Norton [13], in side-coupled waveguide–cavity systems by Fan [14], or in PCs by Gao [15].

In the present publication, we report on a Fano resonance occurring in an Al_0.2_Ga_0.8_N/GaN-based quantum cascade (QC) structure. By smartly exploiting a pronounced quantum-confined Stark effect (QCSE)—thereby partially screening the strong internal piezo- and pyroelectric fields occurring in III-nitride quantum wells (QWs)—we were able to spectrally displace the broad emission of an inter-subband (ISB) transition ‘across’ the nearly constant LO-phonon. Although such Fano-type resonances can theoretically be observed either on the low- or the high-energy side of an ISB peak, we detected the spectrally narrow peak always on the low-energy side of the broader emission peak—along with a decreasingly intense high-energy tail. Using the theoretical analysis of Ugo Fano, we could estimate Fano’s asymmetry parameter ‘q’ being present in our experiments. A careful comparison with previously obtained measurements resulted in a value of q ≈ 4.0.

## 2. Materials and Methods

The III-nitride-based QC layers for these experiments were grown on a C-face sapphire substrate using metal–organic vapor phase epitaxy (MOVPE). On top of a 5 µm-thick n-doped GaN buffer layer (Si, 5 × 10^17^ cm^−3^), a 0.156 µm-thick Al_0.2_Ga_0.8_N/GaN-based active region consisting of 5 periods of a partly n-doped, chirped superlattice with a nominal thickness of 312 Å/period, and finally a 0.2 µm-thick highly n-doped GaN contact layer (Si, 1 × 10^19^ cm^−3^) were grown. In the following Table 1, the (designed) vertical layer structure of the investigated samples (B1767a2 and B1976a1) is shown. As mentioned, the active region of B1767a2 is a superlattice with the following simulated layer sequence: (**25**/33/**15**/22/**12**/20/**14**/**17**/**15**/**15**/**15**/**14**/**18**/13/**21**/11/**22**/10 Å). The first barrier layer is the injection barrier; numbers in plain text indicate pure GaN quantum well (QW) layers, boldface numerals refer to Al_0.2_Ga_0.8_N barrier layers, and underlining stands for n-doping (Si, 5 × 10^17^ cm^−3^). The second sample, B1976a1, had a slightly modified layer sequence (**29**/25/**15**/19/**9**/17/**10**/**15**/**11**/**14**/**12**/**12**/**15**/**10**/**18**/9/**19**/8 Å), which was adapted to its nominally higher barrier layer [Al] content (Al_0.4_Ga_0.6_N), much higher n-type doping (Si, 7 × 10^18^ cm^−3^), and four instead of only three doped layer pairs. Except as explicitly mentioned, we state nominal and not experimentally obtained [Al] contents/layer thicknesses.

In order to measure the actually grown layer thicknesses and compositions, high-resolution X-ray diffraction was performed on each layer structure. In sample B1767a2, for instance, we found that the following slightly deviating layer parameters apply. The active region’s actual period was Λ_SL_ = 361 Å instead of the designed 312 Å (i.e., 15.7% too large), and the actual [Al] content of the Al_x_Ga_1-x_N layers was rather 24.5% instead of the designed 20%. Finally, the (calculated) total ISB energy difference due to the compositional and the thickness deviations in B1767a2 only slightly overcompensated each other and led to a main transition energy being not too far from its design value. A similar, slightly different set of compositional ([Al] content = 23.5% instead of the designed 40%) and thickness (superlattice period Λ_SL_ = 253 Å instead of the designed 267 Å) values was found for layer B1976a1. Compared with the design, a 3.5% too high [Al] composition, along with a 5.24% reduced layer thickness, was determined. But unfortunately, the specific growth conditions of B1976a1—such as high reactant partial pressures/high NH_3_ flow/elevated growth rate along with low gas velocity—‘fostered’ oligomeric pre-reactions. This parasitic effect has probably led to a less efficient incorporation of [Al] atoms into the AlGaN/GaN SL. In the X-ray diffraction data, this effect did not show up prominently because the lower [Al] concentration was ‘compensated’ by larger total AlGaN layer thicknesses. In the end, we could, therefore, have obtained a smaller [Al] content in the AlGaN barriers, a smaller conduction band discontinuity—but thicker AlGaN layers. Since the SL period was nearly at its design value, this resulted in thinner GaN layers. For this reason, the QW states got pushed up partially into the (upper) triangular section. These experimental facts led to a considerably reduced ISB-transition energy, which needed only a small screening voltage to Stark-shift it into resonance with the GaN-based LO-phonon. At the same time, the differential resistance became much smaller, which was observed experimentally as well. The main reason for this reduced resistance was the much smaller remaining barrier height of the involved higher-order excited states—due to a much higher doping.

## 3. Theoretical Aspects

**Figure 1:**
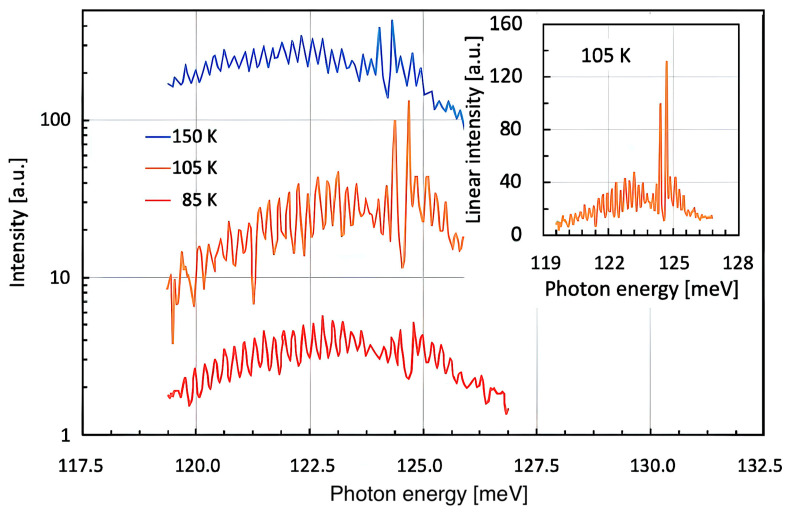
Experimental results of a slightly detuned InGaAs/InAlAs-based QC-DFB laser working at roughly 125 meV (1000 cm^−1^, 10 µm) at device temperatures of 85 K, 105 K, and 150 K. By changing the device temperature, the semiconductor gain peak of this QC structure tunes its spectral position (121.26, 122.13, 122.50 meV (~988 cm^−1^)) more rapidly than the Bragg resonance (123.25, 123.62, 123.74 meV (~998 cm^−1^)). Therefore, an increasing amount of asymmetry is seen at higher temperatures. The inset shows the 105 K-curve in linear scale—thus even better highlighting the spectral asymmetry.

Although the experimental results published by Hans Beutler [5]—being theoretically analyzed by Ugo Fano back in 1935 [2]—triggered a certain academic interest, it took a full 26 years from this initial kickstart until Fano wrote a more comprehensive article about the effect bearing his name until today [6]. As he outlined in his longer publication, ‘Fano resonances’ occur whenever a spectrally narrow feature interacts with a spectrally broad one [16,17]. A typical situation of this kind can be observed in slightly detuned distributed feedback (DFB) lasers, such as shown in Figure 1. We fabricated the mid-infrared QC DFB laser of this particular example in 1999 [18,19,20]. Its ISB luminescence with a relative spectral width of Δν/ν ≈ 16% constitutes the spectrally broad emission, while the Bragg resonance—well visible via the stopband of its optical diffraction grating with Δν/ν ≈ 1%—defines the spectrally narrow emission. Depending on the relative alignment of the two spectra, the narrow peak can occur on either side, as well as right in the center of the broad one, generally resulting in an asymmetric Fano-type luminescence characteristics [21,22,23]. As an interesting side remark, we note here that in semiconductor DFB lasers, the phenomenon ‘Fano resonance’ must thus have been around since 1972 already—although it might not have been described and named as such. In any case, 1972 marks the year when the first DFB lasers were theoretically described by Kogelnik and Shank [24], Henry [25], and experimentally realized by Shank [26], Schinke [27], Nakamura [28], Scifres [29], and Stoll [30]. Since the broad spontaneous emission can be temperature-shifted at a roughly three times higher tuning rate than the narrow Bragg resonance, the degree of asymmetry can be easily adjusted. In agreement with this, we refer to Figure 1, which shows an experimentally measured series of sub-threshold emission spectra from a 10 µm QC DFB laser operated in pulsed mode at 85 K, 105 K, and 150 K.

The experimental spectra shown above are taken from our own publication from 1999 [18]. It is obvious that the Bragg resonance—marked by a double peak with a 0.27 meV (2.2 cm^−1^) wide stopband in between—tunes with temperature at a lower rate of ~7.75 µeV/K, while the ISB gain peak tunes at a roughly 2.5x higher rate of ~19.25 µeV/K. This means that the spectral positions of the two resonances can be adjusted relative to each other by changing the device temperature [31].

**Figure 2:**
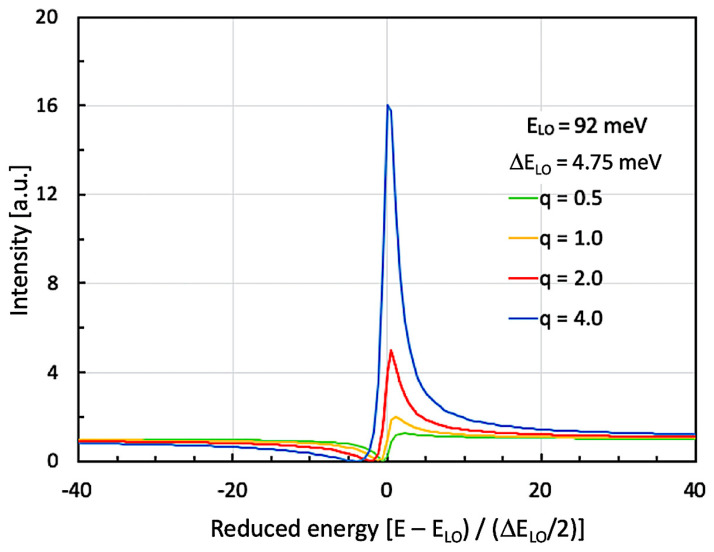
Numerically computed Fano resonance curves for different asymmetry parameters ‘q’. In agreement with the experimentally observed parameters, the GaN-based LO-phonon resonance is located at E_LO_ = 92 meV (741 cm^−1^) and has an FWHM of (ΔE_LO_ = 4.75 meV (38 cm^−1^). The graphs correspond to Fano resonance curves defined by σ(ε) = σ_0_ (q + ε)^2^/(1 + ε^2^) with the ‘reduced energy’ ε = (E − E_LO_)/(ΔE_LO_/2) and σ_0_ being a constant set to σ_0_ = 16.

When now turning back to our QC structure in the III-nitride material system, we first need to calculate how much asymmetry can be expected in the situation described below. For this purpose, we set the narrow resonance at constant energy, namely at the GaN-based LO-phonon at 92 meV (741 cm^−1^). Its width is tentatively defined as 4.75 meV (38 cm^−1^), and we show calculations for four different asymmetry parameters ‘q’. As will be explained below, the spectrally broad feature is given by several overlapping ISB transitions. In order to correctly compare the actually measured luminescence curves with the numerically obtained spectra, we already calculate such Fano resonances for different asymmetry parameters. In our case, this q-parameter is determined by interference between the GaN-based LO-phonon and the slightly detuned overlap of several higher-order ISB transitions. In Figure 2, we present a numerical calculation of a small series of Fano interferences around 92 meV (741 cm^−1^). In this graph, the Fano asymmetry parameter ranges from q = 0.5 up to q = 4.0.

For the comparison with the experimental curves, the x-axis had to be rescaled from ‘normal’ to ‘reduced’ photon energy. By using this figure, we were able to compare these theoretical asymmetries with the experimentally obtained ones. A simple, home-built ‘least-squares-fit’ using the constants E_LO_ = 92 meV, ΔE_LO_ = 4.75 meV, and σ_0_ = 16 resulted—along with an asymmetry parameter q ≈ 4.0 ± 0.5—in the best fit of our LO-phonon luminescence curves. A more detailed analysis is shown in the following paragraph after the description of the experimental results.

## 4. Experimental Results

**Figure 3:**
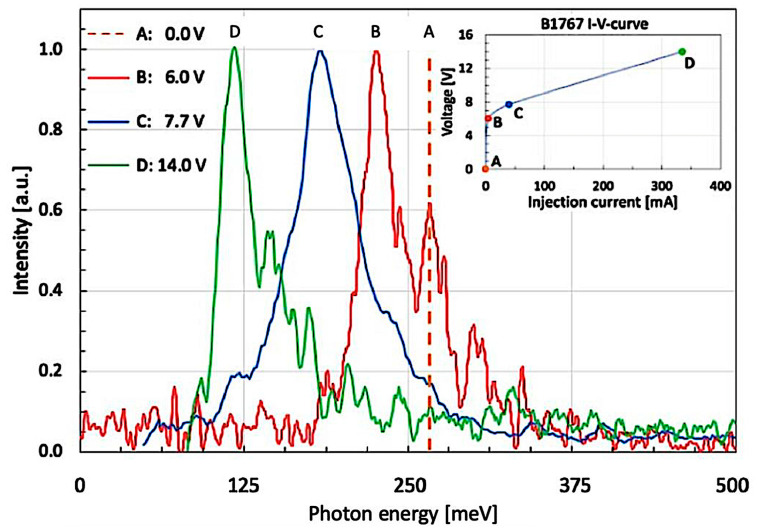
Spectral responses of a d = 38 Å wide and V_0_ = 417 meV deep Al_0.25_Ga_0.75_/GaN-based QW under different pump conditions. Orange (A): hypothetical position of ISB peak at zero applied bias (0 mA/0.0 V). Red (B): electro-modulated absorption (5 mA/6.0 V). Blue (C): weak emission. (40 mA/7.7 V). Green (D): strong emission (335 mA/14.0 V). The inset shows the I–V curve of this device along with the four measured injection current/voltage points.

In the experimental results shown in Figure 3 of the present paper, the spectrally narrow feature—instead of the sharp Bragg resonance of a DFB laser—is played by the LO-phonon peak. The spectrally broad one, on the other hand, is provided by several mutually overlapping higher-order ISB transitions. In our particular experimental setup, all required conditions for the observation of a Fano resonance are thus perfectly met: The GaN-based LO-phonon at an energy of 92 meV (741 cm^−1^) is as narrow as 4.75 meV (38 cm^−1^), broadened by its ultrashort excited state lifetime only [32]. The ISB emission (given, for instance, by the red curve ‘B’), however, is as broad as 62.5 meV (500 cm^−1^). While an LO-phonon would normally result in a symmetric and strictly Lorentzian-shaped absorption or emission peak, our case behaves somewhat differently. Because of the largely different voltage tuning rates of LO-phonon (at nearly constant energy) and ISB luminescence, the latter can be Stark-tuned from a larger energy at 228 meV (1835 cm^−1^—red curve ‘B’) towards a smaller one at 115 meV (926 cm^−1^—green curve ‘D’). When increasing the voltage bias even more, the steep low-energy shoulder of the ISB peak reaches the LO-phonon energy at 92 meV (741 cm^−1^), thus fulfilling a resonance condition and leading to the pronounced LO-phonon peak at 92 meV (741 cm^−1^).

As we have described in our earlier paper [33], the dominant LO-phonon peak at 92 meV (741 cm^−1^) is indeed the result of a resonance phenomenon between the GaN-based LO-phonon and a voltage-tunable, Stark-shifted Al_0.2_Ga_0.8_N/GaN-based ISB emission. In addition, a somewhat closer look reveals that the LO-phonon fulfills two clearly distinct tasks here. At first, it defines the spectral position of the narrow peak at 92 meV (741 cm^−1^). As a second function, it eventually interacts with the broad ISB emission.

Where does the asymmetric ‘foot’ on the high-energy side of the main peak come from? Actually, there are two different experimental situations to be considered here: one with an external voltage ≤ 14 V and another one with a voltage ≥ 18 V, which will be shown later. In the depicted QW with a moderately high (V ≤ 14 V) applied bias voltage, there is a relatively obvious explanation for the observed asymmetry: this ‘foot’ is a clear signature for electrons having undergone higher-order ISB transitions.

Since the externally applied, moderately high electric field is by far not sufficient to entirely screen the internal pyro- and piezo-electric fields, the first excited QW state (E_1_) will be kind of ‘stuck’ in its screening-induced downward movement. However, at the same time, the barrier on the right side keeps moving upwards to higher energies. This way, E_1_ nevertheless ends up slightly below the onset of the triangularly shaped AlGaN barrier, and the fundamental ISB transition (E_1_→E_0_)—which ‘started’ as a quasi-continuum-to-bound transition—becomes of the type ‘bound-to-bound’ with its narrow, much more symmetric spectral characteristics. In Figure 3, this corresponds to the experimental situation C’. As a consequence of this, the broadened higher-order states (E_n_ with n ≥ 2) will all be located within the triangular section of the main QW—where they mutually overlap to form a quasi-continuum. Their increasing diagonality results in this asymmetric high-energy ‘foot’.

Especially at an even higher applied voltage bias/current injection level, there occurs—in contrast to the higher energy ‘foot’ shown in Figure 3—a different, even more pronounced type of spectral asymmetry. When, for example, applying a voltage of 18.5 V onto sample B1767a2, then a narrow luminescence peak at the energy of the LO-phonon at 92 meV (741 cm^−1^) dominates the emission spectrum. This LO-phonon peak is kind of asymmetrically ‘sitting’ on top of a broader, low-intensity peak. At slightly higher energies, however, there is a striking spectral minimum, followed on its high-energy side again by an asymmetric foot. In order to make sure that the observed asymmetry is not just an artifact of one particular epitaxial growth, we investigated devices from later growth runs nominally having either an identical or a very similar device architecture/layer structure.

**Figure 4:**
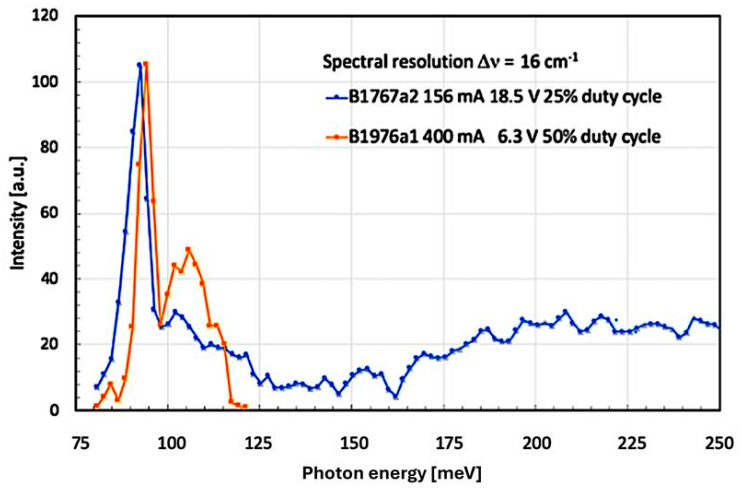
Optical emission under electrical injection as a function of photon energy for two different devices (B1767a2 and B1976a1). Especially in the overview spectrum of B1767a2, the asymmetrically located luminescence peak starting from the frequency of the GaN-based LO-phonon at 92 meV (741 cm^−1^) is clearly visible.

One of these later QC structures, namely sample B1976a1, had been grown nearly one year after B1767a2. Nevertheless, as Figure 4 below shows, the energy of the LO-phonon-based emission peak in this second Al_0.2_Ga_0.8_N/GaN-based QC layer is, at 93.6 meV (755 cm^−1^) almost identical to the one found in B1767a2 at 92 meV (741 cm^−1^). The small difference in the LO-phonon peak energy might be caused by heating or compositional fluctuation effects.

Concerning the spectral differences in the smaller, secondary maximum of the broad luminescence peak (located close to the spectral minimum at 120 meV (960 cm^−1^) for both B1767a2 and B1976a1), we find several possible explanations. First of all, the electrical pump conditions, such as current, voltage, and duty cycle, were not at all identical for the two samples. But even more important is the fact that—as a second point—the mounting of the processed semiconductor chips using a thin [In] foil pressed onto a copper heatsink was not such a well-controlled and established standard procedure. This resulted, without any doubt, in slightly different device temperatures. These multiple factors can, therefore, easily explain the observed small energy difference.

In the curves displayed in Figure 4, we make two additional important observations: Firstly, the LO-phonon peak consistently occurs at the low-energy side of the underlying ISB luminescence. This fact is especially important when turning back to Figure 3: already when the voltage bias of B1767a2 was set to 14.0 V, there existed already a tiny amount of ISB luminescence at the LO-phonon frequency of 92 meV (741 cm^−1^)—located at the low-energy side of the ISB transition! But as Figure 4 shows, sample B1767a2 appears to ‘wait’ until the ISB luminescence has—under the action of a higher applied voltage of 18.5 V—Stark-shifted to a sufficiently small energy. Only at this moment does the resonance with the LO-phonon become precise enough to manifest itself spectrally. As a second important point, this resonance with the asymmetrically broadened ISB luminescence peak is visible for a relatively narrow range of bias voltages only: at voltages <14.0 V, no LO-phonon peak could be measured at all. At 14.0 V, the LO-phonon peak was still very small. It became, however, very large at 18.5 V but already started to fade away at 20.0 V. Finally, at 21.5 V, no trace of an LO-phonon peak was visible anymore. Such a narrow range of strong interaction is a fairly typical property of a resonance—having a high-quality factor. Despite a considerably different total electric field in the two investigated samples (E_tot_ = 0.75 MV/cm for B1767a2 and 1.19 MV/cm for B1976a1—due to a much smaller screening), very closely resembling resonance phenomena have been observed. Based on the specified growth recipe of B1976, a large intentional [Si] doping density (n_3D_ = 7 × 10^18^ cm^−3^) was used. Additionally, at each GaN/AlGaN-based QW/barrier interface, the already quite elevated intentional sheet carrier density (n_s_ = n_3D_ × t = 7 × 10^12^ cm^−2^) was doubled by equally high piezo- and pyro-electric polarizations. In total, we therefore observed n_s_ = 1.4 × 10^13^ cm^−2^, which resulted in a Fermi energy of E_F_ = 170 meV. In addition, the general growth conditions were such as to foster the occurrence of oligomeric pre-reactions, leading overall to a less efficient incorporation of [Al] atoms into the barrier layers. The resulting smaller Al content also decreased not only the conduction band discontinuity but also the AlGaN growth rate. This is qualitatively reflected by a measured Al content of 23.5% instead of the designed Al_0.4_Ga_0.6_N barrier layers and a slightly reduced SL period of 253 Å instead of 267 Å. These two conditions are well reflected by the small differential resistance of B1976a1: at a bias of 6.3 V, a large current of 400 mA was seen. In sample B1767a2, an elevated voltage of 18.5 V was necessary to drive a much smaller current of 156 mA through the sample.

Indeed, a total of 10 nm instead of only 7 nm (per SL period) is doped, leading to a nominal doping (sheet carrier) density of 7 × 10^12^ cm^−2^. Without presenting more detailed numbers, this effect has potentially led to strong band-filling—rendering the ground state—and thus the lowest ISB transition—inaccessible. The next higher ISB transition is, therefore, partially located in the triangular section of the main QW. As a final result, its transition energy is sufficiently small to become resonant with the LO-phonon already at small screening voltage of 6.3 V.

The fact that the low-energy shoulder of our spectrally broadened and asymmetrically shaped ISB luminescence overlaps with the narrow LO-phonon is a characteristic sign of a Fano resonance [2]. The reason for the initially small signal at 92 meV (741 cm^−1^) increasing to a more than ten times higher intensity under the application of just a 32% higher bias voltage of 18.5 V (like Figure 4 for sample B1767a2) is an additional resonance between the LO-phonon and the total layer thickness of the device’s active region. The LO-phonon energy of 92 meV (741 cm^−1^) corresponds to an optical wavelength of 13.5 µm. In the epitaxial layer stack—which consists mainly of GaN with a refractive index of 2.4—the above vacuum wavelength is equivalent to a material wavelength of 13.5 µm/2.4 = 5.625 µm. As the total thickness of the epitaxial layer stack is, at 5.5 µm, just 2.3% smaller than the previously cited value, there is almost perfect resonance between the LO-phonon peak and the total thickness of the epitaxial layers. Since we finally deal here with a conversion from horizontally propagating, TM-polarized electromagnetic ISB radiation—via the scalar quantity ‘energy of an excited LO-phonon’—towards a TE-polarized surface emission, the polarization selection rule for ISB transitions does apparently not strictly apply in the present case. We finally notice a small energy shift between the two samples, B1767a2 and B1976a1. This shift has its origin mainly in the different electric pump power levels and the specific heatsinking conditions of each sample. While B1767a2 received 7.2 W, a mere 1.26 W—along with an improved heatsinking—was applied to B1976a1.

## 5. Numerical Analysis

**Figure 5:**
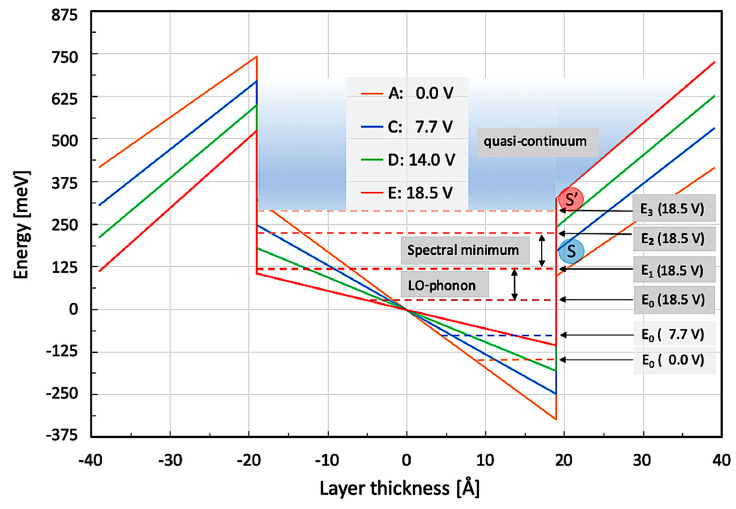
Numerical simulation of the main QW under different external bias voltages with sample B1767a2. For voltages ≤ 14.0 V (blue potential with blue ‘corner’ S), only weak to moderate screening occurs. Energies E_1_ and higher are already located within the triangular QW section. For voltages ≥ 18.5 V (red curve), strong screening occurs. In this case, E_2_ is still confined within the parallel QW section (red potential with red ‘corner’ S’). Due to the relatively large separation between E_1_ and E_2_, a spectral minimum with a width of roughly 200 cm^−1^ occurs between them. Energy levels ≥ E_3_ are contained within the asymmetric part of the QW.

In the following paragraph, we are going to perform some numerical simulations to better understand the experimentally observed external screening of the internal polarization fields. Figure 5 shows the main 38 Å QW of structure B1767a2 alongside its most important bound energy levels under various externally applied bias voltages. As presented in Figure 4, the most important features are the energy difference between E_0_ and E_1_ (becoming resonant to the LO-phonon at 18.5 V), the spectral minimum between 125 meV and 200 meV (being found in the emission spectrum in Figure 4), and the quasi-continuum starting from E_2/3_. This quasi-continuum is due to several closely lying, broadened, and thus overlapping subband states in the triangularly shaped QW starting already above 175 meV at 7.7 V but only above 275 meV at 21.5 V. On the energy scale used in Figure 5 (measured from the center of the active QW) and at a small applied voltage of 7.7 V, the right barrier is obviously much lower than with a large bias voltage of 18.5 V. The concomitant screening of the internal electric polarization fields leads to the occurrence of a spectral asymmetry at considerably smaller energy—in agreement with the asymmetric emission spectra ‘B’, ‘C’, and ‘D’ shown in Figure 3.

Concerning the screening voltages used in Figure 3 and Figure 4, it is important whether the first excited state E_1_ is still below (configuration S)—or already above (configuration S’)—the ‘corner’ (at x = 19 Å) between QW and the adjacent lower-lying barrier (on the ‘right’ side of the QW). At lower bias voltages (≤14.0 V, for instance, in the potential drawn in blue), point S is located below E_2_ so that all higher excited states (E_2_, E_3_, E_4_, E_5_, etc.) will be confined by the triangular part of the QW.

In this context, it is worth noting that in a purely triangular confinement situation, the bound states obey Airy functions, with eigenvalues becoming closer at higher energies, leading to a spectrally asymmetric quasi-continuum. In contrast, in a rectangular quantum well (having infinitely high barriers), the energies of the eigenvalues increase with n^2^ (n being the quantum number). Since, however, the conduction band discontinuity of the rectangular QW is not infinitely high, the upper states condense to a quasi-continuum. In our samples, we have a combination of both: The bottom of the QW is triangular and has an increasing width; the higher part is rectangular, and its conduction band discontinuity is finite: So, the lowest eigenvalues have relatively big Airy differences, becoming smaller at higher energies, having then tendency to become larger at increasing energies due to the rectangular potential shape and decrease in spacing again due to the finite depth of the rectangular QW. Finally, the uppermost part of the QWs has a triangularly widening shape again so that the QW states will condense here as well—to reach a quasi-continuum.

The situation changes, however, for higher applied bias voltages (≥18.5 V). This configuration (S’) is shown as a red potential in Figure 5. The strong screening of the internal polarization field lifts the right barrier of the main QW (at an x-coordinate of 19 Å) sufficiently high so that the lowest QW-based ISB transition (E_1_→E_0_) becomes of the type ‘bound-to-bound’ (configuration S’). At the same time, the transition energy is resonant to the LO-phonon. The spectrally asymmetric emission under higher bias (≥18.5 V) consists, therefore, of the ‘remaining’ mutually overlapping ISB transitions (E_3_→E_0_), (E_4_→E_0_), (E_5_→E_0_), etc., between excited states in the triangular part of the main QW (with n ≥ 3) and the ground state (E_0_). Therefore, the spectral asymmetry shown in Figure 4 can—together with the sharp LO-phonon peak—be interpreted as a classical Fano resonance.

At this point, we compare the numerically computed Fano resonance curves of Figure 2 with the experimental results in Figure 4. When performing this comparison using a least-squares fit, we will be able to estimate a numerically obtained Fano parameter ‘q’. The knowledge of this parameter is necessary for a correct description of the obtained results [34].

**Figure 6:**
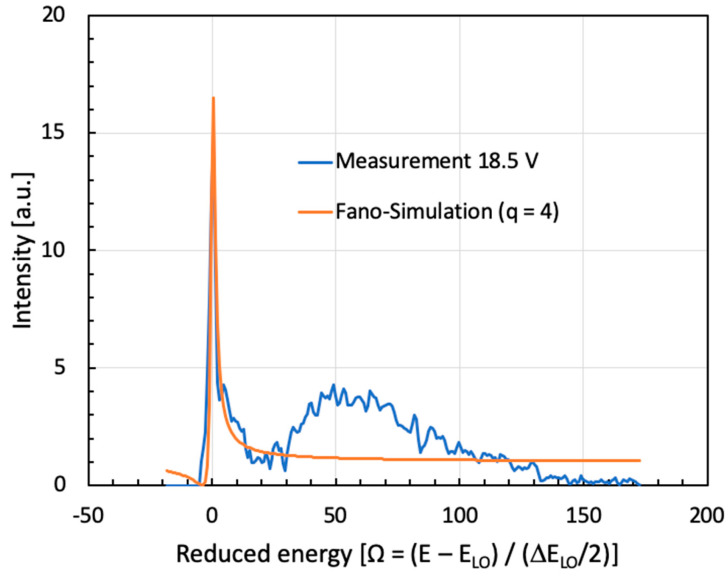
Numerical simulation of the asymmetry parameter ‘q’ present in the measured spectrum of sample B1767a2 at 18.5 V. In our example, the orange curve is a simulation using q = 4.0, E_0_ = 92 meV (741 cm^−1^), and Γ = 4.75 meV (38 cm^−1^). Except for the lowest one (E_1_→E_0_), all other ISB transitions involving excited energies (E_2_→E_0_), (E_3_→E_0_), (E_4_→E_0_), etc., are partly localized in the triangularly shaped main QW of this structure and strongly contribute to the Fano line profile. In order to not account for higher order ISB transitions (i.e., other than E_1_→E_0_), a restricted fitting range between −10 and +30 reduced energy units (‘Ω-units’) was chosen.

Due to the strong screening of the internal field in the main QW, the energetic separation of its two lowest excited states (E_1_ and E_2_) is large enough (>25 meV) to prevent a mutual overlap of the corresponding ISB transitions. As a result of this, there occurs in Figure 4 and Figure 6 kind of a ‘spectral minimum’—located around 140 meV (1100 cm^−1^, i.e., at 20 reduced energy units).

Obviously, one could ask the question of whether a Fano resonance involving the LO-phonon and even higher excited triangular QW states would be possible as well. The answer is ‘yes’. However, we have to take into account that already the resonance with the lowest excited state (E_1_) necessitated a relatively large applied voltage of 18.5 V. In order to Stark-tune an even higher-order ISB transition into resonance with the LO-phonon, a considerably larger voltage of roughly 35–40 V would have to be applied. Whether our samples would have withstood the necessary high electric fields is still an open question. However, it becomes clear from Figure 2 in our *IEEE Photonics Technology Letters* article from 2019 that a maximum of roughly 30 V could be applied to our structure [35]. At voltages exceeding 35 V, the main QW would start to get tilted towards the inverse sense, and the energy difference between the quantized states E_2_ and E_0_ would start to increase again—without ever having reached the energy of the LO-phonon. Anyway, it is surprising that there is even coupling between ISB transitions and optical phonons in the nonpolar material Si. For the highly polar GaN material system, however, it is kind of obvious that such coupling can occur.

## 6. Results and Possible Applications

**Figure 7:**
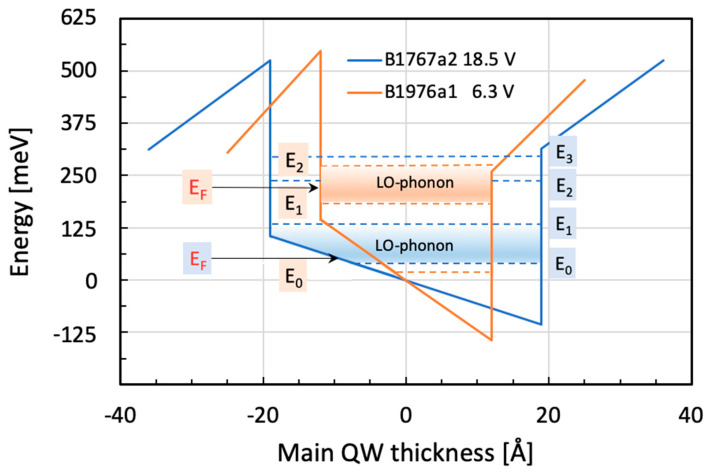
Comparison of the relevant energy levels of samples B1767a2 and B1976a1 showing the same resonance with the LO-phonon. In the 1st sample (B1767a2), the LO-phonon became resonant with the lowest possible E_0_→E_1_ ISB transition. In the 2nd sample (B1976a1), the LO-phonon became resonant with the next higher-order ISB transition, E_1_→E_2_. The respective Fermi levels are shown in red color. All energies are either drawn in blue or in orange according to the band structure.

As the main results, we would like to draw the attention of the readers again to Figure 4, where the emission spectra for three different bias voltages are shown. In all of them, an obvious asymmetry towards higher energies can be seen. This asymmetry is the result of the interference of two differently broad Lorentz-shaped emissions [2,6]. Based on the particular spectral width—measured on either side of the medium line—their asymmetry factor ‘q’ can be determined. It tells us clearly that the observed emission is indeed highly asymmetric—pointing towards the fact that there is a non-negligible degree of interaction between the two types of emission here [15]. In reference [12], for instance, the authors write that their results have significant implications for electromagnetic metamaterials and photonic devices, with potential applications in exotic dispersion modulation and synthesis of light. In Figure 7 below, we compare the band structure of the main QW of the two samples of this study at LO-phonon resonance. It is obvious that the resonance condition was in one case (B1767a2) fulfilled between states E_0_ and E_1_, while in the other case (B1976a1), it occurred between states E_1_ and E_2_. The reason for this marked difference is the much higher doping of the second sample.

It is finally interesting to observe under which name the general topic of a narrow, and a broad resonance mutually interfering with each other has been described already in 1935 [36]. Over the years, the term ‘Fano resonance’ became known also as ‘bound states in the continuum’ [37] or as ‘Fano resonance in photonic crystals’ [8,11].

## 7. Conclusions and Outlook

We have demonstrated a pronounced Fano interference in an Al_0.2_Ga_0.8_N/GaN-based QC emitter. The resonance feature is the result of a strong interaction between the GaN-based LO-phonon at 92 meV and the structure’s spectrally overlapping, broad ISB transitions. An asymmetry parameter of q ≈ 4.0 can be estimated from the observed emission spectrum. Given this strong asymmetry, more precise measurements would result in a much clearer picture of this experimental situation. As some recent literature shows, Fano resonances are also very useful for areas such as optical switching and sensing, or electromagnetically induced transparency [38]. Additional examples of such potential applications include papers about Fano resonances in a broader context by Limonov [39] or Chu [40]; about ‘Fano resonances in epsilon-near-zero media’ by Yan [41]; or in an overview article of ‘bound states in the continuum’ by Hsu [42].

The examples cited here stand for the widespread scientific interest in Fano resonances [43,44]. However, they also prove that the Fano theory is a quite universal effect having applications in very diverse fields [45]. With the demonstration of new device concepts such as photonic crystals [11,15], Fano resonances have obviously experienced a veritable revival [46].

## 8. Patents

Since none of the contributing authors is currently working in the area of Fano resonances in the III-nitride semiconductors, there are no patent issues associated with this research.

## Figures and Tables

**Table 1:** Generic layer descriptions, materials, thicknesses, and doping levels of the proposed QC structures (B1767a2/B1976a1).

Layers	Materials	Thicknesses	Si Doping Levels
Cap layer	GaN	0.200 µm	1 × 10^19^ cm^−3^
Active region B1767 or active region B1976	GaN/Al_0.2_Ga_0.8_N or GaN/Al_0.4_Ga_0.6_N	0.156 µm or 0.134 µm	5 × 10^17^ cm^−3^ or 7 × 10^18^ cm^−3^
Buffer layer	GaN	5.000 µm	5 × 10^17^ cm^−3^
Substrate	Al_2_O_3_	−	undoped

## Data Availability

The original data are available from D.H. on request.
